# Photobiomodulation Enhances the Angiogenic Effect of Mesenchymal Stem Cells to Mitigate Radiation-Induced Enteropathy

**DOI:** 10.3390/ijms20051131

**Published:** 2019-03-05

**Authors:** Kyuchang Kim, Janet Lee, Hyosun Jang, Sunhoo Park, Jiyoung Na, Jae Kyung Myung, Min-Jung Kim, Won-Suk Jang, Sun-Joo Lee, Hyewon Kim, Hyunwook Myung, JiHoon Kang, Sehwan Shim

**Affiliations:** 1 Laboratory of Radiation Exposure & Therapeutics, National Radiation Emergency Medical Center, Korea Institute of Radiological and Medical Sciences, Seoul 01812, Korea; kimkyuch@gmail.com (K.K.); lys5017@kirams.re.kr (J.L.); hsjang@kirams.re.kr (H.J.); sunhoo@kirams.re.kr (S.P.); njy0914@kirams.re.kr (J.N.); tontos016@kirams.re.kr (J.K.M.); kimmj74@kirams.re.kr (M.-J.K.); wsjang@kirams.re.kr (W.-S.J.); sjlee@kirams.re.kr (S.-J.L.); hw0227@kirams.re.kr (H.K.); doctor_myung@daum.net (H.M.); jhkang4293@gmail.com (J.K.); 2 Department of Pathology, Korea Cancer Center Hospital, Korea Institute of Radiological and Medical Sciences, Seoul 01812, Korea; 3 Department of Veterinary Surgery, College of Veterinary Medicine, Konkuk University, Seoul 05029, Korea

**Keywords:** photobiomodulation, umbilical cord blood-derived mesenchymal stem cell, angiogenesis, radiation-induced enteropathy, paracrine effect

## Abstract

Radiation-induced enteropathy remains a major complication after accidental or therapeutic exposure to ionizing radiation. Recent evidence suggests that intestinal microvascular damage significantly affects the development of radiation enteropathy. Mesenchymal stem cell (MSC) therapy is a promising tool to regenerate various tissues, including skin and intestine. Further, photobiomodulation (PBM), or low-level light therapy, can accelerate wound healing, especially by stimulating angiogenesis, and stem cells are particularly susceptible to PBM. Here, we explored the effect of PBM on the therapeutic potential of MSCs for the management of radiation enteropathy. In vitro, using human umbilical cord blood-derived MSCs, PBM increased proliferation and self-renewal. Intriguingly, the conditioned medium from MSCs treated with PBM attenuated irradiation-induced apoptosis and impaired tube formation in vascular endothelial cells, and these protective effects were associated with the upregulation of several angiogenic factors. In a mouse model of radiation-induced enteropathy, treatment with PBM-preconditioned MSCs alleviated mucosal destruction, improved crypt cell proliferation and epithelial barrier functions, and significantly attenuated the loss of microvascular endothelial cells in the irradiated intestinal mucosa. This treatment also significantly increased angiogenesis in the lamina propria. Together, we suggest that PBM enhances the angiogenic potential of MSCs, leading to improved therapeutic efficacy for the treatment of radiation-induced enteropathy.

## 1. Introduction

The intestine is one of the most vulnerable organs to radiation toxicity due to its rapid proliferation [1]. Radiation-induced enteropathy can be induced either in cancer patients undergoing abdominopelvic radiotherapy or in individuals exposed to accidental irradiation. The early and late adverse effects of radiation in the intestine not only impair quality of life, but can also be life-threatening in some cases [2,3]. Unfortunately, few effective treatments exist, and clinical applications are often limited due to a lack of efficacy or unfavorable side effects [4]. Therefore, there is an urgent need to develop medical countermeasures that are effective in treating radiation-induced enteropathy.

Regarding the “target cell” for the treatment of acute radiation-induced injury to the bowel, the depletion of progenitor epithelial cells in the crypts of Lieberkühn has long been considered the only determinant of early toxicity [5]. The contemporary understanding of the pathogenesis of radiation-induced enteropathy is far more complex, and involves interactions among multiple cell compartments including epithelial, mesenchymal, and vascular endothelial cells, as well as the immune system, the enteric nervous system, and the gut microbiome [2]. Among these cell populations, considerable evidence has indicated that the endothelium plays a central role in normal tissue injury induced by radiation exposure. For example, microvascular endothelial cell apoptosis precedes crypt stem cell damage after irradiation [6,7]. In addition, the prevention of radiation-induced endothelial damage attenuates crypt cell loss and dysfunction, organ failure, and death [8,9,10,11].

Mesenchymal stem cell (MSC)-based therapy has the potential to induce angiogenesis, mainly via the secretion of angiogenic growth factors. It was shown that the paracrine properties of MSCs can enhance collateral vessel growth in ischemic tissue, bone regeneration, cardiovascular repair following myocardial infarction, and wound healing [12,13,14,15]. Unfortunately, limitations still remain concerning the clinical application of MSCs, which is primarily due to their low therapeutic efficacy. Specifically, most of the applied MSCs are removed from the body within one week of transplantation. In fact, the transplanted MSCs themselves do not undergo angiogenesis, but rather paracrine factors released from the transplanted cells are actually responsible for stimulating host angiogenesis [16]. To overcome this limitation of stem cell therapy, preconditioning, genetic modification, and the optimization on MSC culture conditions have been explored in the field of tissue engineering and regenerative medicine [17].

Photobiomodulation (PBM), which is commonly referred to as low-level laser/light therapy, is the therapeutic application of light. This treatment affects endogenous chromophores in the body to stimulate non-harmful and non-thermal reactions at the cellular level that result in a beneficial therapeutic outcome [18]. Recently, PBM treatment was suggested to stimulate new blood vessel growth. Visible red to near-infrared light in the wavelength range of 600 to 1000 nm was found to enhance tissue healing by stimulating angiogenesis in various animal models of ischemia [19]. Moreover, stem cells and progenitor cells appear to be particularly susceptible to PBM [20]. Indeed, this approach has shown the potential to promote MSC proliferation, differentiation, and growth factor secretion [21,22]. However, the effect of PBM-preconditioned MSCs on radiation-induced intestinal injury has not been investigated.

Therefore, the present study aimed to investigate whether PBM preconditioning enhances the therapeutic effects of MSCs for radiation-induced enteropathy, focusing on the role of the endothelial cell compartment.

## 2. Results

### 2.1. PBM Enhances the Proliferation and Self-Renewal of MSCs

**Figure 1:**
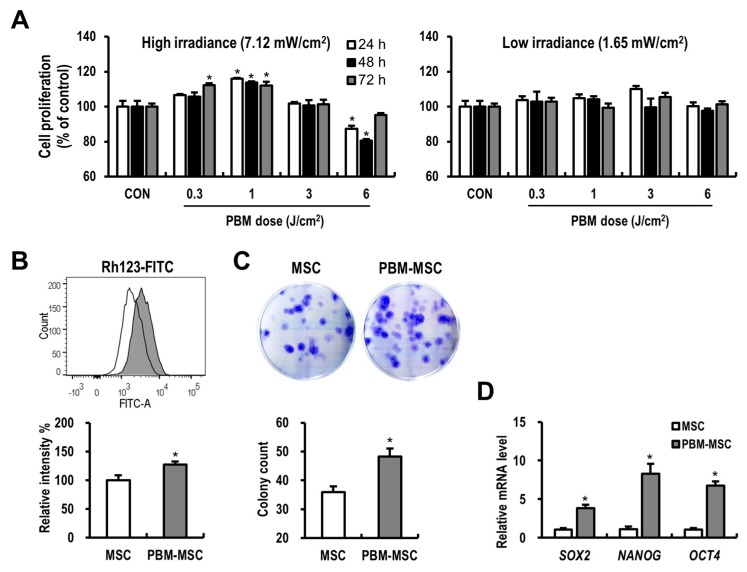
Photobiomodulation (PBM) enhances the proliferation and self-renewal of mesenchymal stem cells (MSCs). (**A**) Cell proliferation assays using MSCs treated with or without PBM at the indicated light parameters. As an irradiance of 7.12 mW/cm^2^ and a fluence of 1 J/cm^2^ were considered most beneficial to stimulate MSC proliferation, the following experiments were conducted using this condition. (**B**) Mitochondrial membrane potential of unconditioned MSCs (MSC; open histogram) and PBM-treated MSCs (PBM-MSC; filled histogram), as detected by rhodamine 123 (Rh123) fluorescence. (**C**) Clonogenic colony-forming unit-fibroblast (CFU-f) assay for MSC and PBM-MSC groups based on 0.5% crystal violet staining. (**D**) Relative mRNA expression of stemness-related genes in MSCs analyzed by real-time reverse transcription-polymerase chain reaction (RT-PCR). Data are presented as the mean ± standard error of the mean (SEM); *n* ≥ 3 per group. * *p* < 0.05 compared to the control.

To assess whether PBM affects MSC proliferation and to determine the dose required for maximal enhancement of MSC ability, we used variable fluences. PBM at 1 J/cm^2^ with high irradiance resulted in a significant increase in MSC proliferation compared to that in control cells at 24 h, 48 h, and 72 h post-treatment (Figure 1A). It is possible that the dose rate and the total energy dose delivered could affect the outcome, and thus we next evaluated the effect of low irradiance, wherein cells were placed farther from the light source and with a longer duration of exposure to achieve the same fluences. Low-irradiance PBM slightly increased MSC proliferation, as compared to that in control cells, but this effect was not significant. Accordingly, 1 J/cm^2^ with high irradiance was selected as the optimal dose for the further experiments. As the treatment of PBM increases the membrane potential of mitochondria, which is a parameter of mitochondrial function in cells [20,23], we assessed the mitochondrial activity in MSCs after PBM application using a Rhodamine 123 (Rh123) assay (Figure 1B). The mean fluorescence intensity of Rh123 in MSCs was significantly increased in the PBM-treated group compared to that in the control group. To examine the effect of PBM on the long-term expansion of MSCs, a colony-forming unit-fibroblast (CFU-f) assay was performed (Figure 1C). Consistent with the results of short-term proliferation assays, PBM-treated MSCs formed more colonies than control cells after 10 days of culture. Furthermore, PBM treatment induced the upregulation of stemness-related genes, including *sex determining region Y-box 2* (*SOX2*), *nanog homeobox* (*NANOG*), and *octamer-binding transcription factor 4* (*OCT4*) in MSCs (Figure 1D). These data suggested that PBM improves the proliferative properties and self-renewal capacity of MSCs.

### 2.2. PBM Maintains the Immunophenotype and Differentiation Potential of MSCs

**Figure 2:**
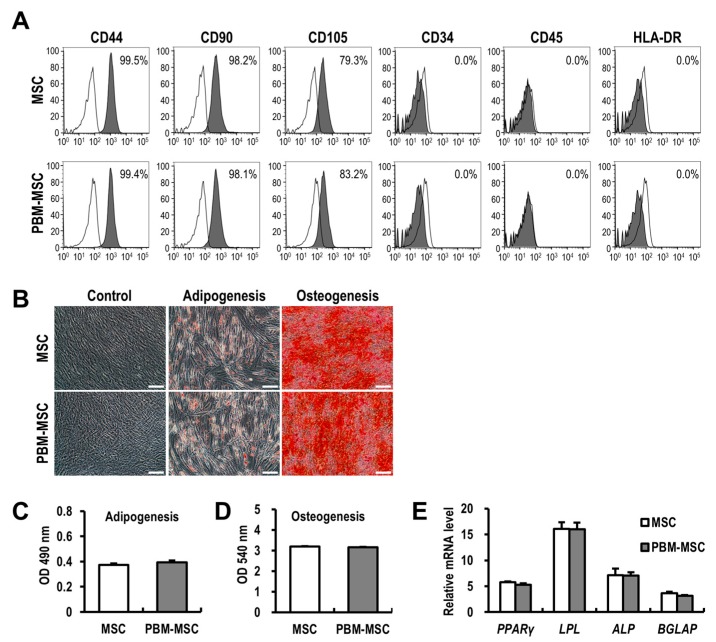
Photobiomodulation (PBM) maintains the immunophenotype and differentiation potential of mesenchymal stem cells (MSCs). (**A**) The immunophenotype of unconditioned MSCs (MSC) and PBM-treated MSCs (PBM-MSC). Percentage of positively-labeled cells for each surface marker (filled histogram) compared to an isotype control (open histogram), as determined by flow cytometry. (**B**) Representative images of undifferentiated MSCs and those differentiated into adipogenic or osteogenic lineages, treated with or without daily PBM. Scale bar = 100 μm. The degree of differentiation was quantified by measuring absorbance (**C**,**D**), and by the real-time RT-PCR analysis of differentiation markers (**E**). The mRNA expression levels are shown relative to those in undifferentiated controls as one. Data are presented as the mean ± SEM; *n* ≥ 3 per group.

The three minimal standard criteria proposed by the International Society of Cellular Therapy (ISCT) to define MSCs include: (i) adherence to plastic; (ii) expression of typical cell surface molecules; and (iii) tri-lineage differentiation potential in vitro. Here, the flow cytometric analysis of immunophenotypes showed high similarity between PBM-treated and control MSCs with respect to positive [cluster of differentiation (CD)44, CD90, and CD105] and negative [CD34, CD45, and human leukocyte antigen-DR isotype (HLA-DR)] marker expression (Figure 2A). To investigate whether PBM affects the differentiation potential of MSCs, adipogenic and osteogenic differentiation were visualized using specific stains after 14 days of induction (Figure 2B). Daily treatment of MSCs with PBM over 14 days resulted in no difference in the extent of adipogenic and osteogenic differentiation, as compared to that in untreated cells (Figure 2C,D). In addition, mRNA levels of markers of adipogenesis [*peroxisome proliferator-activated receptor gamma* (*PPARγ*) and *lipoprotein lipase* (*LPL*)] and osteogenesis [*alkaline phosphatase* (*ALP*) and *bone gamma-carboxyglutamate protein* (*BGLAP*)] were not different between PBM-treated and control MSCs (Figure 2E). Taken together, these data suggested that the application of PBM maintains the essential properties of MSCs.

### 2.3. PBM Promotes the Angiogenic Capacity of MSCs to Attenuate Radiation-Induced Damage to Vascular Endothelial Cells

**Figure 3:**
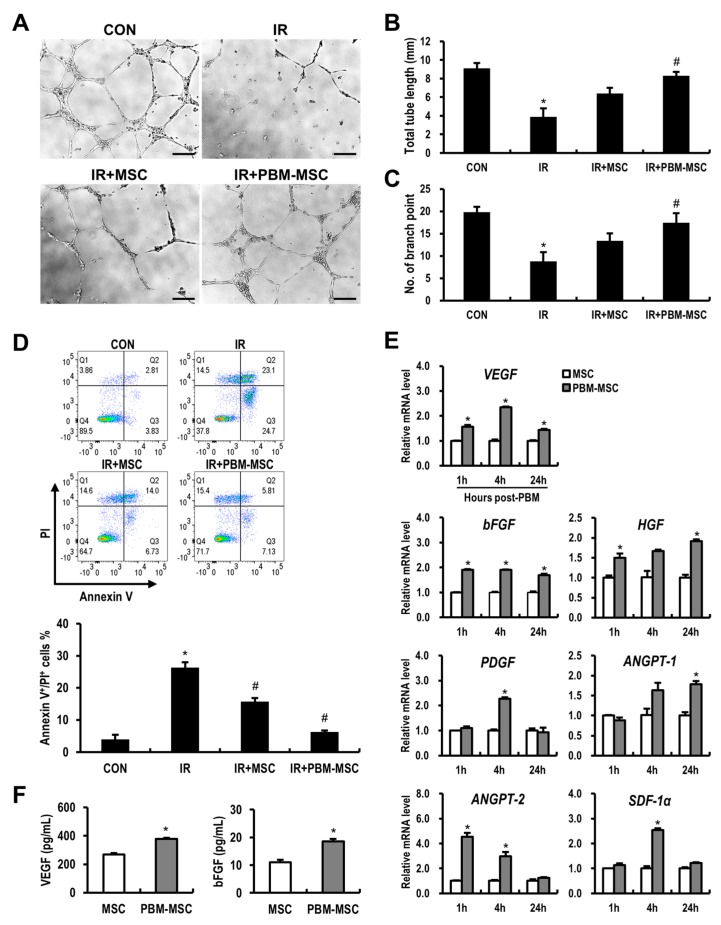
Photobiomodulation (PBM) promotes the angiogenic capacity of mesenchymal stem cells (MSCs) to reduce radiation-induced damage to vascular endothelial cells. (**A**) Capillary-like tube formation assays using human umbilical vein endothelial cells (HUVECs) are grouped as follows: control (CON), irradiated (IR), IR and MSC-conditioned medium (CM) treatment (IR+MSC), IR and PBM-preconditioned MSC-CM treatment (IR+PBM-MSC). Scale bar = 200 μm. (**B**) Total tube length and (**C**) the number of branch points were measured in each group. (**D**) HUVEC apoptosis was analyzed by flow cytometry. The percentage of apoptotic cell death was defined as double-positivity for Annexin V and propidium iodide (PI). (**E**) mRNA levels of angiogenesis-related genes in unconditioned MSCs (MSC) and PBM-preconditioned MSCs (PBM-MSC) at the indicated time points. (**F**) Concentrations of vascular endothelial growth factor (VEGF) and basic fibroblast growth factor (bFGF) in the MSC-CM and PBM-MSC-CM, as determined by enzyme-linked immunosorbent assay (ELISA). Data are presented as the mean ± SEM; *n* ≥ 3 per group. * *p* < 0.05 compared to the control; ^#^ *p* < 0.05 compared to the IR group.

Endothelial cells are considered a prime target of radiation-induced toxicity to normal tissue, including the intestine [6]. We also identified that radiation exposure induces impaired angiogenesis in human umbilical vein endothelial cells (HUVECs) based on tube formation assays (Figure 3A). Moreover, with irradiated HUVECs, the PBM-preconditioned MSC-conditioned medium (MSC-CM) group showed a significant increase in total tube length and the number of branch points compared to those in the IR group (Figure 3B,C). Next, we investigated the protective effects of PBM-preconditioned MSC-CM with respect to radiation-induced endothelial apoptosis (Figure 3D). As shown in Figure 3D, MSC-CM treatment decreased the proportion of Annexin V and propidium iodide (PI)-double positive irradiated HUVECs. In addition, HUVEC apoptosis was further reduced by PBM-preconditioned MSC-CM treatment. MSCs synthesize a diverse array of cytokines, some of which greatly affect endothelial survival, growth, and angiogenesis [12]. Using real-time reverse transcription-polymerase chain reaction (RT-PCR), we examined the effect of PBM on proangiogenic gene expression in MSCs (Figure 3E). We found that PBM upregulated a subset of angiogenesis-related genes, including *vascular endothelial growth factor* (*VEGF*), *hepatocyte growth factor* (*HGF*), *basic fibroblast growth factor* (*bFGF*), *platelet-derived growth factor* (*PDGF*), *angiopoietin* (*ANGPT*)*-1*, *ANGPT-2*, and *stromal cell-derived factor 1 alpha* (*SDF-1α*). In addition, concentrations of VEGF and bFGF in the PBM-preconditioned MSC-CM were significantly higher than those in the MSC-CM, as determined by enzyme-linked immunosorbent assay (ELISA) (Figure 3F). Together, these data suggested that preconditioning MSCs with PBM enhances angiogenesis by inhibiting endothelial apoptosis and accelerating angiogenic factor production. 

### 2.4. PBM Preconditioning Enhances the Therapeutic Efficacy of MSCs against Radiation-Induced Enteropathy

**Figure 4:**
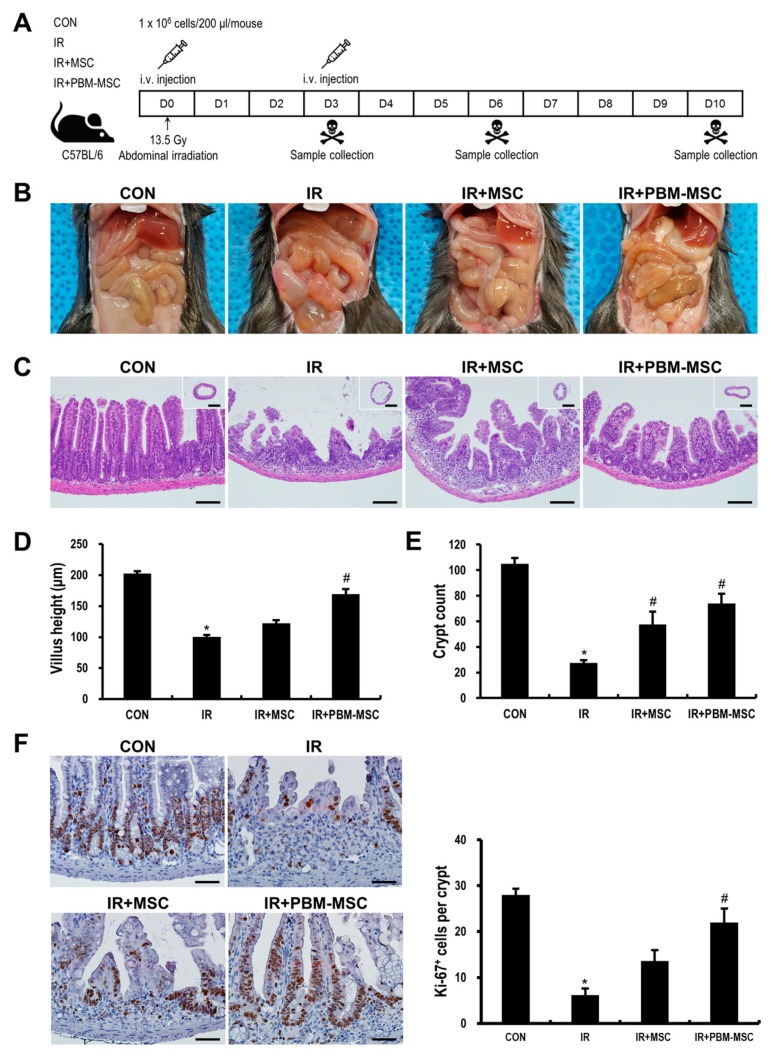
Photobiomodulation (PBM) preconditioning enhances the therapeutic efficacy of mesenchymal stem cells (MSCs) against radiation-induced enteropathy. (**A**) Schematic diagram of the in vivo experimental protocol. Mice were exposed to a single dose of 13.5-Gy x-ray irradiation administered to the whole abdomen and then treated with an intravenous injection of MSCs (IR+MSC), PBM-preconditioned MSCs (IR+PBM-MSC), or vehicle (IR) twice, with a two-day interval. (**B**) Representative images of gross pathology at Day 6 post-irradiation. (**C**) Hematoxylin and eosin (H&E) staining of the small intestinal tissue in control (CON), IR, IR+MSC, and IR+PBM-MSC groups. Scale bar = 100 μm for main images; 1 mm for inserts. Quantification of (**D**) villus height and (**E**) crypt count per intestinal circumference. (**F**) Immunohistochemical analysis of Ki-67 staining in the small intestinal tissue, representing epithelial cell proliferation. Scale bar = 50 μm. Data are presented as the mean ± SEM; *n* = 5 per group. * *p* < 0.05 compared to the control; ^#^ *p* < 0.05 compared to the IR group.

The in vivo experimental schedule is presented in Figure 4A. Mice were exposed to a single dose of 13.5 Gy administered to the whole abdomen under anesthesia. Two hours after irradiation, MSCs (IR+MSC), PBM-preconditioned MSCs (IR+PBM-MSC), or vehicle [phosphate-buffered saline (PBS); IR] was intravenously injected into irradiated mice, which was followed by a second injection 2 days later. At 6 days after irradiation, a time point at which the symptomatic and histological abnormalities were most severe in our experimental setting, gross pathology showed that the intestinal content became watery upon irradiation, and this pathological change was attenuated in the IR+PBM-MSC group (Figure 4B). Histological analysis revealed that the crypt–villus units of the intestinal mucosa were severely destroyed in the IR group, as evidenced by the flattened villi and decreased number of surviving crypts (Figure 4C–E). In contrast, the loss of villi and crypts was mitigated by MSC treatment, and these mucosal structures were further maintained in the IR+PBM-MSC group. In addition, the number of proliferating epithelial cells, which was represented by Ki-67 expression, was significantly increased in the IR+PBM-MSC group as compared to that in the IR group (Figure 4F). At Day 10 post-irradiation, the villus height of the IR group was restored near to normal, but the crypts were still fewer and appeared enlarged and distorted (Figure S1A–S1C). These abnormalities were not seen in the IR+PBM-MSC group. In addition, a decrease in the body weight after irradiation recovered faster in the IR+PBM-MSC group, although it was not statistically significant compared to the IR group (Figure S1D). Symptoms of acute radiation enteropathy include diarrhea and fluid loss [2]. We excluded loose or watery feces from the cage bedding and counted the well-formed feces for each group. A higher number of formed feces was found in the IR+PBM-MSC group than those in the IR or IR+MSC group (Figure S1E). To confirm that the therapeutic effects of PBM-preconditioned MSCs depend on their paracrine activity, we also conducted experiments using MSC-CM. Histological analysis at Day 6 revealed that the histological damage after irradiation was attenuated by daily treatment with PBM-preconditioned MSC-CM, but not by treatment with MSC-CM or intermittent treatment with PBM-preconditioned MSC-CM (Figure S2A–S2C). Taken together, the paracrine effect of MSCs is reinforced by PBM preconditioning to attenuate radiation-induced damage to the intestine.

### 2.5. PBM-Preconditioned MSCs Attenuate Intestinal Barrier Damage and Inflammation During Radiation-Induced Enteropathy

**Figure 5:**
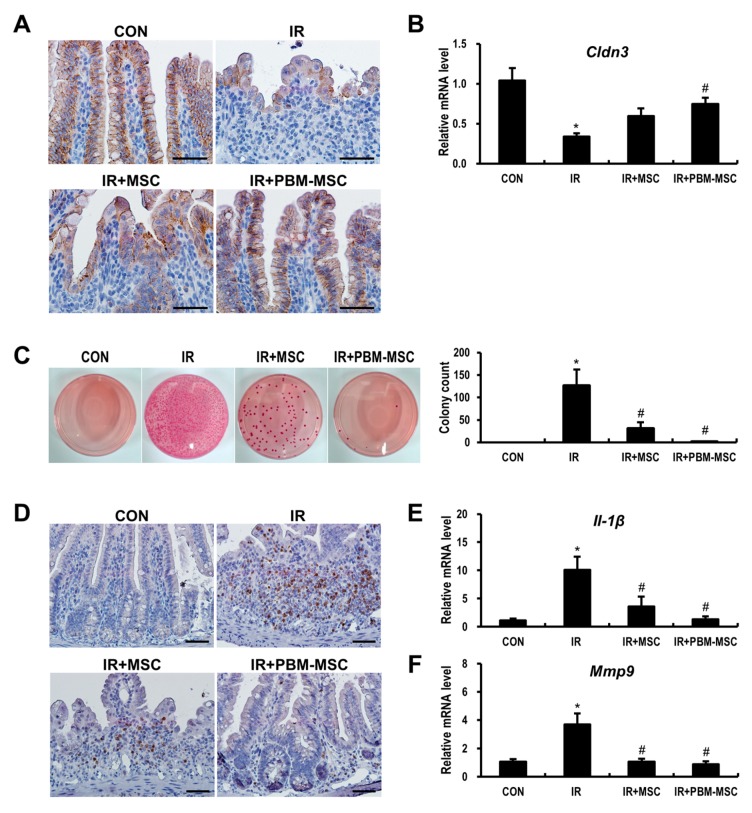
Photobiomodulation (PBM)-preconditioned mesenchymal stem cells (MSCs) attenuate intestinal barrier damage and inflammation during radiation-induced enteropathy. (**A**) Immunohistochemical analysis and (**B**) mRNA levels of claudin 3 (Cldn3) in the small intestinal tissue of control (CON), irradiated (IR), IR and MSC-treated (IR+MSC), and IR and PBM-preconditioned MSC-treated (IR+PBM-MSC) groups. Scale bar = 50 μm. (**C**) The number of bacterial colonies isolated from mesenteric lymph node tissue. (**D**) Immunohistochemical analysis of myeloperoxidase (Mpo) and mRNA expression of (**E**) *interleukin* (*Il*)-1*β* and (**F**) *matrix metallopeptidase* (*Mmp*)*9* in the small intestinal tissue of CON, IR, IR+MSC, and IR+PBM-MSC groups. Scale bar = 50 μm. Data are presented as the mean ± SEM; *n* = 5 per group. * *p* < 0.05 compared to the control; ^#^ *p* < 0.05 compared to the IR group.

The disruption of epithelial integrity can lead to intestinal barrier dysfunction, which facilitates the parenteral access of enteric bacteria, thereby causing sepsis and even death [24]. As the epithelial integrity is controlled by multiple intercellular tight junction (TJ) molecules, we compared the expression of claudin 3 (Cldn3), which is one such TJ protein. The irradiated intestine showed weak immunoreactivity for Cldn3 when compared to that in controls (Figure 5A). However, this reduced Cldn3 expression was rescued by MSC treatment. Notably, the immunoreactivity of Cldn3 was further improved in the IR+PBM-MSC group, resulting in near-normal staining. In addition, the mRNA level of Cldn3 was significantly decreased following irradiation (0.4-fold compared to control levels), which was somewhat normalized by PBM-MSC treatment in the irradiated intestine (Figure 5B). We also evaluated bacterial translocation to the mesenteric lymph nodes, which is the first-pass organ encountered by translocating microorganisms (Figure 5C). Whereas the samples from control mice were sterile, a large number of colonies were observed in the IR group. Moreover, colony counts were significantly decreased in the IR+PBM-MSC group compared to that in the IR group. Next, we elucidated the anti-inflammatory effects of PBM-preconditioned MSCs using intestinal tissue exhibiting radiation-induced enteropathy. The accumulation of neutrophils in the intestine corresponds to the severity of inflammation in radiation enteritis [25]. Cells positive for myeloperoxidase (Mpo), which is a marker of activated neutrophils, were significantly increased in the intestine of the IR group compared to that in the control group (Figure 5D). However, PBM-MSC treatment attenuated the immunoreactivity of Mpo in the irradiated intestine. Interleukin (Il)-1β and matrix metallopeptidase (Mmp)9 expression are also markedly increased during acute inflammation after radiation exposure [26]. Based on our data, Il-1β and Mmp9 expression were significantly increased in the intestines of the IR group, whereas the levels of these inflammatory cytokines were decreased in the IR+PBM-MSC group (Figure 5E,F). Collectively, these data suggested that PBM-preconditioned MSCs attenuate intestinal barrier dysfunction and the inflammatory response during radiation-induced enteropathy.

### 2.6. PBM-Preconditioned MSCs Rescue Microvasculature Damage in the Irradiated Intestine

**Figure 6:**
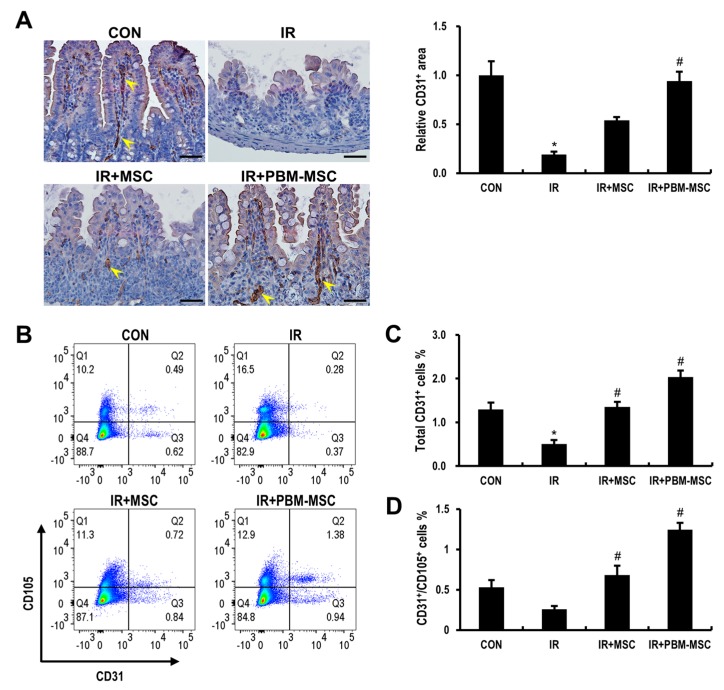
Photobiomodulation (PBM)-preconditioned mesenchymal stem cells (MSCs) restore the microvasculature of the irradiated intestine. (**A**) Immunohistochemical analysis of CD31 in the small intestinal tissue of control (CON), irradiated (IR), IR and MSC-treated (IR+MSC), and IR and PBM-preconditioned MSC-treated (IR+PBM-MSC) groups. Scale bar = 50 μm. Arrowheads indicate a CD31-positive area. (**B**) Flow cytometric analysis to enumerate the endothelial cells in the isolated lamina propria of CON, IR, IR+MSC, and IR+PBM-MSC groups. (**C**) Total endothelial cells in the isolated lamina propria were determined by CD31-positive cells. (**D**) Angiogenesis in the isolated lamina propria was determined by CD31 and CD105-double positive cells. Data are presented as the mean ± SEM; *n* ≥ 3 per group. * *p* < 0.05 compared to the control; ^#^ *p* < 0.05 compared to the IR group.

As our in vitro data indicated the pro-angiogenic effects of PBM-treated MSCs on irradiated endothelial cells, we identified the therapeutic effects of these cells based on the improvement of angiogenetic defects during radiation-induced enteropathy. To evaluate microvascular endothelial cells in the intestine, immunohistochemical staining for CD31, which is a pan-endothelial marker, was performed three days after irradiation (Figure 6A). At this time point, there was a significant decrease in the microvessel density, which was represented by the CD31-positive area, in the IR group compared to that in the control group. Moreover, the CD31-positive area was markedly increased in the intestine of the IR+PBM-MSC group compared to that in the IR group. We also confirmed that the number of CD31-positive endothelial cells in the lamina propria was higher in the IR+PBM-MSC group compared to that in the other groups based on flow cytometric analysis (Figure 6B,C). CD31 and CD105-double positive cells indicate increased angiogenesis [27,28], which plays a critical role in regenerative therapy. Here, the IR+PBM-MSC group had the highest number of CD31 and CD105-double positive cells in the lamina propria when compared to that in other groups (Figure 6D). Taken together, these data suggested that PBM-preconditioned MSCs improve the restoration of intestinal microvasculature after irradiation.

## 3. Discussion

Over the last decade, a number of preclinical studies have encouraged the application of MSC-based therapies to repair intestinal radiation-induced damage. Such benefits of MSCs include anti-inflammatory effects, the promotion of neovascularization, and the restoration of epithelial integrity [3,29]. However, several hurdles need to be overcome before MSC therapies can be brought into clinical use for patients with radiation-induced enteropathy. Especially, one of the major associated problems is the poor therapeutic efficacy of transplanted MSCs according to the disease microenvironment. Thus, to improve the therapeutic activity of MSCs for tissue damage induced by irradiation, many studies have been performed, including the pre-activation of MSCs with pro-inflammatory molecules, the application of biocompatible scaffolds, and MSC homing-related gene modifications [30,31,32,33]. In this study, we applied PBM to improve the therapeutic effects of MSC and identified that MSCs pre-activated with PBM exert angiogenic effects and mitigate radiation-induced enteropathy.

The therapeutic use of light has been considered a non-invasive modality for clinical applications such as the reduction of inflammation, edema, and pain [34,35,36]. Moreover, the application of PBM, which is a form of phototherapy, in the visible red to near-infrared region of the spectrum (600–1000 nm), has beneficial effects on a variety of diseases and physiological processes, including wound healing, hypoxia injury, and cerebral degeneration [37,38,39]. The effects of PBM are biologically attributed to the absorption of light by the endogenous photoreceptors in the respiratory chain located within the mitochondria and the induction of mitochondrial activation in the cells. Notably, PBM affects stem cells or progenitor cells, enhancing their proliferation and recruitment from circulation [40,41,42]. Yin et al. reported that PBM improves not only proliferation but also the synthesis of growth factors such as HGF and PDGF by regulating mitochondrial activation in MSCs [43]. We also identified that PBM treatment enhances MSC proliferation, which was associated with increased mitochondrial membrane potential and self-renewal capacity, at a dose of 1 J/cm^2^ with high irradiance. In addition, MSCs treated with PBM induced the synthesis of angiogenic growth factors. These data suggested the upregulation of several angiogenic factors following PBM treatment, likely via increases in mitochondrial membrane potential, in MSCs.

We also showed that irradiated HUVECs exhibit a decline in angiogenic ability with increased apoptosis. Moreover, the endothelial damage caused by irradiation induced inflammation and abnormal crypt proliferation. Endothelial injury by irradiation is a key event during the initiation of damage to normal tissues [6,7,10,11]. Extensive damage to microvascular endothelial cells of the lamina propria leads to inflammation and the loss or dysfunction of crypt stem cell clonogens during radiation-induced enteropathy. Moreover, the prevention of endothelial cell damage by growth factors such as VEGF, bFGF, and ANGPT-1 variants inhibit crypt cell damage, organ failure, and death during radiation-induced gastrointestinal syndrome [6,8,9]. MSCs exhibit potent angiogenic growth factor secretion, including VEGF, bFGF, HGF, epidermal growth factor, glial-derived neurotrophic factor, angiogenin, and IL-8 [44]. These paracrine effects have been used to successfully treat several disease models such as periodontal defects, Parkinson’s disease, and diabetes-associated vascular injury [45,46,47]. We also identified that PBM-treated MSCs improve angiogenic effects in irradiated endothelial cells and inhibit endothelial apoptosis by enhancing the MSC secretome including VEGF, bFGF, HGF, ANGPT-1, ANGPT-2, and PDGF. Further, in vivo data showed that PBM-treated MSCs protected against endothelial damage and promote angiogenesis during radiation-induced intestinal injury.

Radiation-induced intestinal injury is associated with intestinal barrier dysfunction and inflammatory reactions with neutrophil infiltration and increased inflammatory cytokines such as IL-1β and MMP9 [26]. Based on our data, neutrophil infiltration and inflammatory cytokines were increased in the irradiated intestinal tissues, and this was associated with bacterial translocation to the mesenteric lymph node. These results suggested that intestinal inflammation and epithelial barrier dysfunction are accompanied by endothelial dysfunction upon radiation-induced intestinal injury. However, PBM-preconditioned MSCs exerted anti-inflammatory effects by attenuating endothelial dysfunction, and also improved intestinal barrier damage.

In summary, the present study revealed the therapeutic effects of PBM-treated MSCs on radiation-induced endothelial injury both in vitro and in vivo. Our findings highlight the pivotal role of the vascular component during radiation-induced intestinal toxicity. Although the exact mechanisms through which therapeutically applied MSCs prevent vascular endothelial cell loss are still unclear, their ability to suppress radiation-induced apoptosis in endothelial cells might be relevant, as this results in improved endothelial cell survival, as shown here and by others. Our results indicate that PBM preconditioning improves the pro-angiogenic capacity of MSCs, thereby alleviating radiation-induced intestinal injury more effectively than MSC treatment alone. Future studies should focus on the potential clinical use (e.g., optimizing the quality, dosing, timing, and delivery strategy of PBM) and further elucidate their mechanism of action to develop innovative medical countermeasures suitable for radiation-induced intestinal injury.

## 4. Materials and Methods

### 4.1. Cell Culture

Human umbilical cord blood-derived MSCs were purchased from MEDIPOST Co., Ltd. (Gyeonggi-do, Korea), and cells at passages three to five were used for experiments. MSCs were cultured in minimum essential medium-alpha (MEM-α; Gibco, Grand Island, NY, USA) supplemented with 10% fetal bovine serum (FBS; Invitrogen, Carlsbad, CA, USA) and 1% penicillin-streptomycin (Gibco). HUVECs (Lonza, Basel, Switzerland) at passages three to five were cultured in endothelial cell basal medium-2 (EBM-2; Lonza) supplemented with endothelial cell growth medium-2 BulletKit (EGM-2; Lonza). Culture media were replaced every three to four days, and cultures were maintained in a humidified incubator at 37 °C with 5% CO_2_.

### 4.2. PBM Preconditioning

MSCs were treated with light generated by a light-emitting diode array (Healite Mini; Lutornic Corp., Gyeonggi-do, Korea). This device was determined to emit 633-nm visible red light. The irradiance (power density) measured with an optical power meter (2832-C; Newport Inc., Irvine, CA, USA) was 7.12 mW/cm^2^ at a distance of 3 cm (high irradiance), and 1.65 mW/cm^2^ (low irradiance) at 10 cm from the light source. Fluence, the total amount of energy delivered per unit area, was defined as the irradiance integrated over time (1 mW/cm^2^ × 1 second = 0.001 J/cm^2^) and referred to interchangeably as “dose”. The duration of exposure was varied to achieve 0 J/cm^2^, 0.3 J/cm^2^, 1 J/cm^2^, 3 J/cm^2^, or 6 J/cm^2^ to treat MSC (Table 1).

### 4.3. Cell Proliferation

Cell proliferation was evaluated using a colorimetric method based on water-soluble tetrazolium salts (WST-1; CellVia, Abfrontier, Seoul, Korea). MSCs were seeded at a density of 5 × 10^3^ cells/well in 96-well culture plates. Then, the cells were treated once with various energy doses of PBM as described. After 24 h, 48 h, or 72 h of incubation, 10 µL of WST-1 was added to the cells, which were incubated for an additional 1 hour at 37 °C. Proliferation was measured using a microplate reader at a wavelength of 450 nm. As MSCs treated with a dose of 1 J/cm^2^ exhibited the highest proliferation, the following experiments were performed with this dose.

### 4.4. Mitochondrial Membrane Potential

To assess the effect of PBM on mitochondrial function, the mitochondrial specific probe Rh123 (Sigma-Aldrich, St. Louis, MO, USA) was used. One hour after PBM treatment, MSCs were harvested and incubated in PBS containing 5 µg/mL Rh123 for 30 min at 37 °C. After washing twice with PBS, the cells were analyzed by flow cytometry (FACSCanto II; BD Biosciences, Franklin Lakes, NJ, USA) using the fluorescein isothiocyanate (FITC) channel.

### 4.5. Colony-Forming Unit-Fibroblast (CFU-f) Clonogenic Assay

MSCs were seeded at a density of 200 cells/well in six-well culture plates and cultured with or without daily PBM treatment. After 10 days of culture, the media were removed, and the cells were washed with PBS and stained with 0.5% crystal violet in methanol for 10 min at room temperature. After repeated rinsing with distilled water, the number of colonies was counted manually. Colonies were defined as isolated groups of at least 10 cells.

### 4.6. Immunophenotype of MSCs

For phenotypic analysis, sham-treated or PBM-treated MSCs were stained with FITC-conjugated, allophycocyanin (APC)-conjugated, or phycoerythrin (PE)-conjugated antibodies specific for human CD34, CD44, CD45, CD90, CD105, and HLA-DR (eBioscience, San Diego, CA, USA) and analyzed by flow cytometry. The cell surface markers were selected according to the standard criteria of the ISCT [48].

### 4.7. Adipogenic and Osteogenic Differentiation of MSCs

MSC were induced to differentiate into adipogenic and osteogenic lineages, as described previously with minor modifications [49]. For osteogenic differentiation, MSCs were plated at 1 × 10^5^ cells/well in six-well culture plates. After 1 day of incubation, the cells were washed in PBS and then cultured in Dulbecco’s modified Eagle medium (DMEM; WELGENE, Daegu, Korea) supplemented with 0.1 μmol/L of dexamethasone, 100 μmol/L of L-ascorbic acid 2-phosphate, 10 mmol/L of β-glycerol phosphate, and 10% FBS. For adipogenic differentiation, MSCs were plated at 5 × 10^5^ cells/well in six-well culture plates. After 1 day of incubation, the cells were washed in PBS and then cultured in Iscove’s modified Dulbecco’s medium (WELGENE) supplemented with 1 μmol/L of dexamethasone, 0.2 mmol/L of indomethacin, 0.5 mmol/L of 3-isobutyl-1-methylxanthine, and 10% FBS. To examine the effect of PBM on MSC differentiation, treatment was performed once per day for 14 days of induction. After the induction period, differentiation was evaluated using Oil Red O staining for adipogenesis and Alizarin Red S staining for osteogenesis. Then, the degree of differentiation was quantified using a microplate reader. In addition, the expression of differentiation markers was also analyzed by real-time RT-PCR.

### 4.8. RNA Extraction and Real-Time RT-PCR

Total RNA was extracted from the intestine tissues of mice using TRIzol reagent (Invitrogen) and from cultured MSCs using the RNeasy Mini Kit (Qiagen, Hilden, Germany). Next, 1 µg of RNA was used for cDNA synthesis with the Accupower RT Premix Kit (Bioneer, Daejeon, Korea) according to the manufacturer’s instructions. Real-time RT-PCR was performed using a LightCycler 480 system (Roche, San Francisco, CA, USA) with SYBR Green (Roche) for the detection of gene expression. The primer sequences used are listed in Table 2. Gene expression data were normalized to the expression levels of *GAPDH* or *β-actin* (ΔCT values). Fold changes were calculated as 2^−ΔΔ*C*t^.

### 4.9. Preparation of MSC-Conditioned Medium

To obtain MSC-CM, 1 × 10^6^ MSCs per flask were seeded in T75 culture flasks. After incubation for 24 h, the attached cells were washed three times with PBS, and the complete medium was replaced with serum-free MEM-α. Following sham or PBM treatment, the cells were incubated for an additional 24 h, and the supernatant was harvested. For HUVEC apoptosis and tube formation assays, EBM-2 supplemented with 2% FBS was used instead of serum-free MEM-α.

### 4.10. HUVEC Apoptosis and Capillary-Like Tube Formation Assays

Subconfluent HUVECs were exposed to 15 Gy of irradiation at a dose rate of 3.25 Gy/min using a ^137^Cs γ-ray irradiator (Gammacell 3000 Elan; MDS Nordion, Ontario, Canada). For apoptosis assays, the complete medium was replaced with EBM-2 with 2% FBS, MSC-CM, or PBM-treated MSC-CM. After 24 h of incubation, apoptosis rates were determined by flow cytometry using Annexin V and PI staining (FITC-Annexin V Apoptosis Detection Kit I; BD), according to the manufacturer’s instructions. For tube formation assays, the irradiated HUVECs were detached and re-plated in Matrigel (Corning, Corning, NY, USA)-coated 24-well culture plates using three types of media (the same as those used for apoptosis assays). After 16 h of incubation, the cells were fixed with 4% paraformaldehyde. Four microscopic fields per group were randomly selected to measure total tube length and count the number of branch points using CellSens software (Olympus, Waltham, MA, USA).

### 4.11. ELISA

To determine the protein levels of growth factors released from MSCs, MSC-CM obtained under serum-free condition was tested using Quantikine ELISA kits for human VEGF and human bFGF (R&D Systems, Minneapolis, MN, USA).

### 4.12. Animals

Six to seven-week-old male specific pathogen-free (SPF) C57BL/6 mice were purchased from DooYeol Biotech (Seoul, Korea) and maintained under SPF conditions at the animal facility of the Korea Institute of Radiological and Medical Sciences (KIRAMS). All of the mice were housed in a temperature-controlled room with a 12-hour light/dark cycle, and food and water were provided ad libitum. The mice were acclimated for 1 week before experiments and assigned to the following groups: (1) control (CON, *n* = 28), (2) irradiation with vehicle (PBS) treatment (IR, *n* = 28), (3) irradiation with MSC treatment (IR+MSC, *n* = 28), and (4) irradiation with PBM-preconditioned MSC treatment (IR+PBM-MSC, *n* = 28). All of the animal experiments were performed in accordance with the guidelines of the Institutional Animal Care and Use Committee of KIRAMS, which also approved this study (Approval number: kirams2018-0058; Approval date: 8 November 2018).

### 4.13. Irradiation and MSC Administration

Animals were anesthetized via the intraperitoneal injection of 85 mg/kg of alfaxalone (Alfaxan; Careside, Gyeonggi-do, Korea) and 10 mg/kg of xylazine (Rompun; Bayer Korea, Seoul, Korea). Then, they were irradiated once via whole-body irradiation at a dose of 13.5 Gy and a dose rate of 2 Gy/min using an X-RAD 320 X-ray irradiator (Precision X-Ray; North Branford, CT, USA). MSCs were prepared on the day of administration. First, MSCs treated with or without PBM were detached using 0.05% trypsin–ethylenediaminetetraacetic acid (EDTA). Trypsinization was performed within 30 min of PBM. After washing three times, the cells were re-suspended in PBS. According to their groups, mice were injected with 1 × 10^6^ cells/200 μL/mouse or the same volume of PBS through the lateral tail vein. The injection was administered 2 h post-irradiation and again on Day 2 after irradiation (Figure 4A).

### 4.14. Histological Analysis

Small intestine samples isolated from mice were fixed with a 10% neutral-buffered formalin solution, embedded in paraffin wax, and sectioned transversely to a thickness of 4 µm. Then, the sections were stained with hematoxylin and eosin (H&E). To perform immunohistochemical analysis, slides were subjected to heat-induced antigen retrieval in tris-EDTA (pH 9.0) buffer and then treated with 0.3% hydrogen peroxide in methyl alcohol for 20 min to block endogenous peroxidase activity. After three washes with PBS, the sections were blocked with 10% normal goat serum (Vector ABC Elite kit; Vector Laboratories, Burlingame, CA, USA) and incubated with anti-mouse Ki-67 (Acris, Rockville, MD, USA), anti-mouse Cldn3 (Invitrogen), anti-mouse Mpo (Abcam, Cambridge, UK), and anti-mouse CD31 (Abcam) antibodies. After three washes with PBS, the sections were incubated with a horseradish peroxidase-conjugated secondary antibody (Dako, Carpinteria, CA, USA) for 60 min. The peroxidase reaction was developed using a diaminobenzidine substrate (Dako), which was prepared according to the manufacturer’s instructions, and the slides were counterstained with hematoxylin. To quantify microvessel density, 10 villi/mouse were evaluated, and the percentage of CD31-positive to total villi area was calculated using Image J (NIH, Bethesda, MD, USA).

### 4.15. Bacterial Translocation

To assess the translocation of enteric bacteria to regional lymph nodes, mesenteric lymph nodes were harvested from mice under sterile conditions on Day 6. An aliquot of each lymph node homogenate was plated on MacConkey agar (BD) and incubated at 37 °C. After 18 h of incubation, the individual colonies were manually counted.

### 4.16. Flow Cytometric Analysis of Isolated Intestinal Stromal Cells

Stromal cells in the lamina propria were isolated from the intestine of mice as previously described with minor modifications [50]. Briefly, a 10-cm long piece of terminal ilium was excised and washed in PBS to remove luminal contents. After the removal of Peyer’s patches from the ileum, the gut fragments were opened longitudinally, cut into 2-cm pieces, and incubated for 20 min at 37 °C in calcium-free and magnesium-free DMEM (Gibco) containing 10 mM of EDTA. The tissue pieces were washed by vortexing three times with Dulbecco’s PBS to obtain a clear supernatant devoid of epithelial cells. Subsequently, the gut pieces were minced into 1-mm fragments and incubated for 20 min at 37 °C in a dissociation mixture composed of 5 mL of DMEM, Liberase TL (1 Wünsch unit/mL; Roche), and DNase I (1 U/mL; Invitrogen). After 20 min, the supernatants were harvested, and one volume of DMEM containing 10% bovine serum (BS; Sigma-Aldrich) was added while adding 5 mL of fresh dissociation mixture to the remaining tissue pieces. This enzymatic digestion was repeated three times for 60 min in total. Next, the remaining intestine fragments were mechanically disaggregated using a 100-μm mesh. The cellular suspension that was obtained was washed twice with DMEM to remove floating debris and filtered twice through a 40-μm mesh. After centrifugation, cells were re-suspended in PBS containing 2% BS and 2 mM of EDTA and subjected to staining with APC or phycoerythrin–cyanine 7 (PE-Cy7)-conjugated anti-mouse CD31 and CD105 antibodies (eBioscience) for flow cytometry.

### 4.17. Statistical Analysis

Data are presented as the mean ± standard error of the mean (SEM). Statistical analyses were performed using one-way analysis of variance (ANOVA) with Tukey’s multiple comparison test. A *p* value < 0.05 was considered statistically significant.

## Figures and Tables

**Table 1:** Light-emitting diode device used for photobiomodulation.

Wavelength	Irradiance (mW/cm^2^)	Duration (sec)	Fluence (J/cm^2^)
633 nm	1.65	182 606 1818 3636	0.3 1 3 6
	7.12	42 140 421 843	0.3 1 3 6

**Table 2:** Primer sequences used for real-time RT-PCR.

Species	Primer	Forward (5′–3′)	Reverse (5′–3′)	bp
Human	SOX2	GCCCTGCAGTACAACTCCAT	GACTTGACCACCGAACCCAT	128
	NANOG	AAGGCCTCAGCACCTACCTA	TGCACCAGGTCTGAGTGTTC	181
	OCT4	GATGTGGTCCGAGTGTGGTT	AGCCTGGGGTACCAAAATGG	160
	PPARγ	CTAAAGAGCCTGCGAAAG	TGTCTGTCTCCGTCTTCTTG	331
	LPL	TCAACTGGATGGAGGAG	GGGGCTTCTGCATACTCAAA	169
	ALP	CAACAGGGTAGATTTCTCTTGG	GGTCAGATCCAGAATGTTCC	135
	BGLAP	GGCAGCGAGGTAGTGAAGAG	CAGCAGAGCGACACCCTAGAC	195
	VEGF	CCCACTGAGGAGTCCAACAT	TTTCTTGCGCTTTCGTTTTT	186
	bFGF	CGACCCTCACATCAAGCTACA	CGTTTCAGTGCCACATACCAA	219
	HGF	ATCAAATGTCAGCCCTGGAG	TCGATAACTCTCCCCATTGC	207
	ANGPT-1	CATTCTTCGCTGCCATTCTG	GCACATTGCCCATGTTGAATC	103
	ANGPT-2	ACTGTGTCCTCTTCCACCAC	GGATGTTTAGGGTCTTGCTTT	132
	PDGF	GCAAGACCAGGACGGTCATTT	GGCACTTGACACTGCTCGT	135
	SDF-1α	TCAGCCTGAGCTACAGATGC	CTTTAGCTTCGGGTCAATGC	161
	GAPDH	GGACTCATGACCACAGTCCATGCC	TCAGGGATGACCTTGCCCACAG	152
Mouse	Il-1β	GGTCAAAGGTTTGGAAGCAG	TGTGAAATGCCACCTTTTGA	94
	Mmp9	GCCCTGGAACTCACACGACA	TTGGAAACTCACACGCCAGAAG	85
	Cldn3	AAGCCGAATGGACAAAGAA	CTGGCAAGTAGCTGCAGTG	72
	β-actin	TCCCTGGAGAAGAGCTATGA	CGATAAAGGAAGGCTGGAA	100

## Supplementary Materials

Supplementary materials can be found at https://www.mdpi.com/1422-0067/20/5/1131/s1.

## Abbreviations

ANGPT	angiopoietin
ANOVA	analysis of variance
APC	allophycocyanin
bFGF	basic fibroblast growth factor
BGLAP	bone gamma-carboxyglutamate protein
CD	cluster of differentiation
CFU-f	colony-forming unit-fibroblast
Cldn3	claudin 3
DMEM	Dulbecco’s modified Eagle medium
EBM-2	endothelial cell basal medium-2
EDTA	ethylenediaminetetraacetic acid
EGM-2	endothelial cell growth medium-2
FBS	fetal bovine serum
FITC	fluorescein isothiocyanate
HGF	hepatocyte growth factor
HLA-DR	human leukocyte antigen-DR isotype
HUVEC	human umbilical vein endothelial cell
H&E	hematoxylin and eosin
Il	interleukin
ISCT	International Society of Cellular therapy
KIRAMS	Korea Institute of Radiological and Medical Sciences
LPL	lipoprotein lipase
MEM-α	minimum essential medium-alpha
Mmp	matrix metallopeptidase
Mpo	myeloperoxidase
MSC	mesenchymal stem cell
MSC-CM	MSC-conditioned medium
NANOG	nanog homeobox
OCT4	octamer-binding transcription factor 4
PBM	photobiomodulation
PBS	phosphate-buffered saline
PDGF	platelet-derived growth factor
PE	phycoerythrin
PE-Cy7	phycoerythrin-cyanine 7
PI	propidium iodide
PPARγ	peroxisome proliferator-activated receptor gamma
Rh123	rhodamine 123
RT-PCR	reverse transcription-polymerase chain reaction
SDF-1α	stromal cell-derived factor 1 alpha
SEM	standard error of the mean
SOX2	sex determining region Y-box 2
SPF	specific pathogen-free
TJ	tight junction
VEGF	vascular endothelial growth factor
