# Supplementary material for: Photobiomodulation Enhances the Angiogenic Effect of Mesenchymal Stem Cells to Mitigate Radiation-Induced Enteropathy
Source: Int J Mol Sci. 2019 Mar 5;20(5):1131. doi: 10.3390/ijms20051131 (PMC6429482; doi:10.3390/ijms20051131)
Supplement: Supplementary file 1 [file ijms-20-01131-s001.pdf]

Supplementary materials

Photobiomodulation Enhances the Angiogenic Effect of Mesenchymal Stem Cells to Mitigate Radiation-Induced Enteropathy

1. The beneficial effects of photobiomodulation (PBM)-preconditioned mesenchymal stem cells (MSCs) are maintained during recovery from radiation-induced enteropathy

Male C57BL/6 mice were exposed to a single dose of 13.5-Gy x-ray irradiation administered to the whole abdomen and then treated with an intravenous injection of MSCs (IR+MSC), PBM-preconditioned MSCs (IR+PBM-MSC), or vehicle (IR) twice, with a 2-day interval. Three mice per group were sacrificed 10 days after irradiation, and small intestinal samples isolated from mice underwent histological analysis. During the experimental period, body weight of each mouse was measured every day. Fecal pellets formed in 24 hours were collected from the cage bedding at Day 2, 5, and 8 post-irradiation. Then the formed feces were counted for each group.

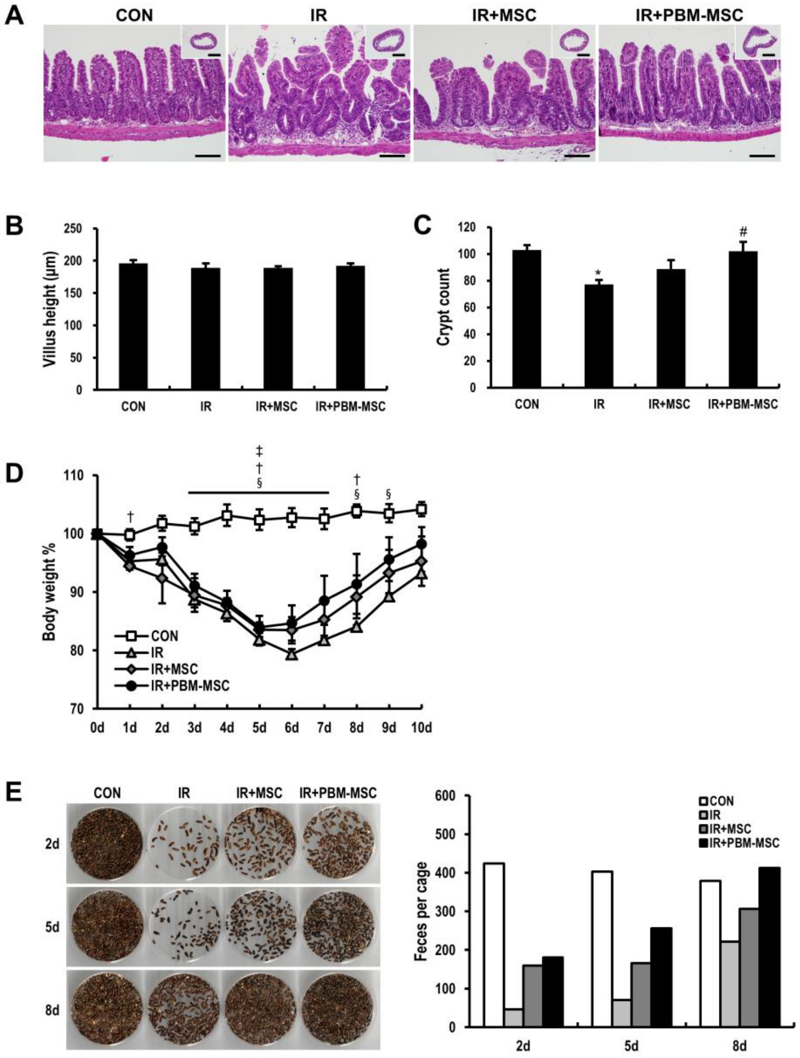

Figure S1. (**A**) Hematoxylin and eosin staining of the small intestinal tissue in control (CON), irradiation with vehicle treatment (IR), irradiation with MSC treatment (IR+MSC), and irradiation with PBM-preconditioned MSC treatment (IR+PBM-MSC) groups at Day 10 post-irradiation. Scale bar = 100 μm for main images; 1 mm for inserts. Quantification of (**B**) villus height and (**C**) crypt count per intestinal circumference. (**D**) Body weight changes of mice in each group. (**E**) Formed feces collected from cage bedding on each day. Data are presented as the mean ± SEM; n = 3 per group. * $p < 0.05$ compared to the control; $^{\#}$ $p < 0.05$ compared to the IR; § $p < 0.05$, CON vs. IR; † $p <$ 0.05, CON vs. IR+MSC; ‡ $p < 0.05$, CON vs. IR+PBM-MSC.

2. Conditioned medium from PBM-treated MSCs mitigates radiation-induced enteropathy

MSCs were plated at a density of 1×10^6 cells/flask in T75 culture flasks. The attached cells were washed three times with phosphate-buffered saline and the complete medium was replaced with 15 mL of serum-free minimum essential medium-alpha (MEM-α). Twenty-four hours after sham or PBM treatment, the conditioned medium (CM) was harvested. Then the sham- or PBM-preconditioned MSC-CM was concentrated 75-fold by ultrafiltration with 3 kDa cut-off (Merck Millipore, Burlington, MA, USA), to obtain 200 μL from each flask. Cell-free MEM-α was also concentrated in the same way to serve as a vehicle control. Male C57BL/6 mice were assigned to the following groups: (1) vehicle (MEM-α) treatment (CON, n = 4), (2) irradiation with vehicle treatment (IR, n = 4), (3) irradiation with MSC-CM treatment (IR+MSC-CM, n = 8), (4) irradiation with PBM-preconditioned MSC-CM treatment (IR+PBM-MSC-CM, n = 8). IR+MSC-CM and IR+PBM-MSC-CM groups were further divided into sub-groups in which CM was administered twice at Day 0 and 2 (IR+MSC-CM-2 or IR+PBM-MSC-CM-2), or 6 times through Day 0 to 5 (IR+MSC-CM-6 or IR+PBM-MSC-CM-6). The vehicle treatment was done through Day 0 to 5. According to their groups, 200 μL of medium per mouse was intraperitoneally administered.

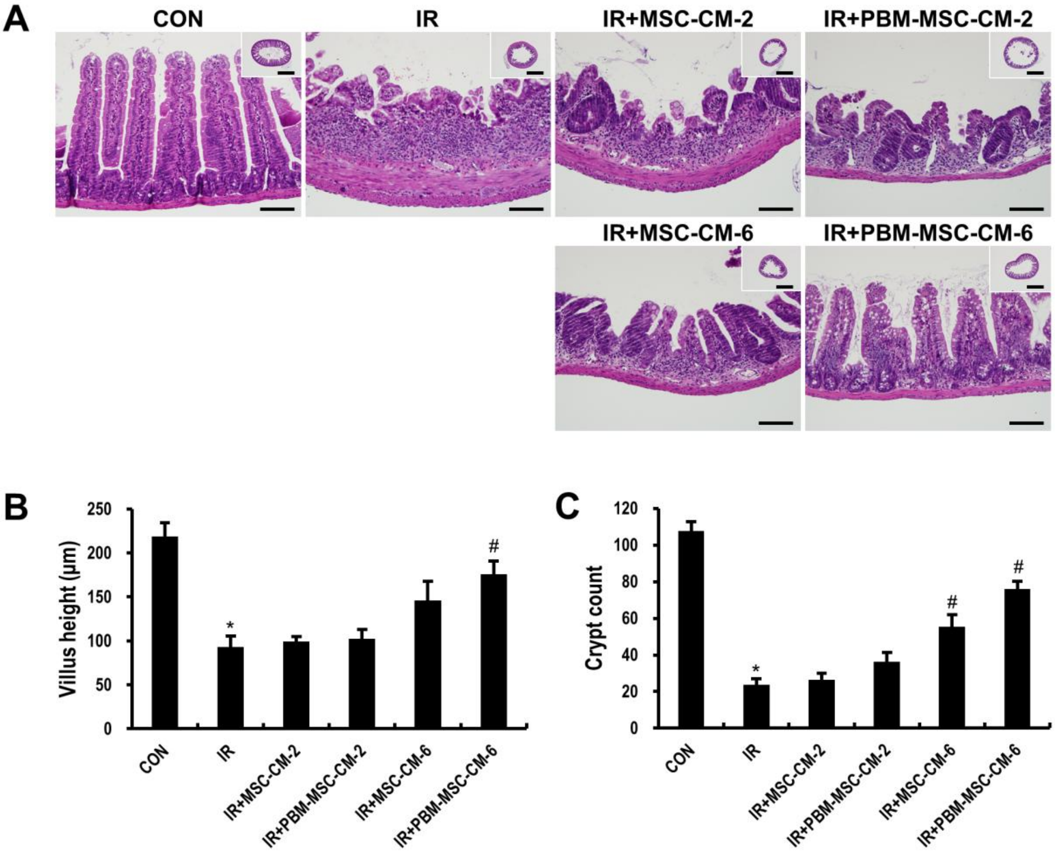

Figure S2. (**A**) Hematoxylin and eosin staining of the small intestinal tissue in control (CON), irradiation with vehicle treatment (IR), irradiation with MSC-CM treatment (IR+MSC-CM), and irradiation with PBM-preconditioned MSC-CM treatment (IR+PBM-MSC-CM) groups at Day 6 post-irradiation. The numbers after the group name indicate the number of treatments. Scale bar = 100 μm for main images; 1 mm for inserts. Quantification of (**D**) villus height and (**E**) crypt count per intestinal circumference. Data are presented as the mean ± SEM; n = 4 per group. * $p < 0.05$ compared to the control; # $p < 0.05$ compared to the IR group.
